# Apremilast or Methotrexate: The Arrows in the Quiver for Psoriasis

**DOI:** 10.7759/cureus.38802

**Published:** 2023-05-09

**Authors:** Gautam Panda, Jyoti Prakash Sahoo, Prasenjeet Mohanty, Trupti R Swain

**Affiliations:** 1 Pharmacology, Sriram Chandra Bhanja Medical College and Hospital, Bhubaneswar, IND; 2 Pharmacology, Kalinga Institute of Medical Sciences, Bhubaneswar, IND; 3 Dermatology, Sriram Chandra Bhanja Medical College and Hospital, Cuttack, IND; 4 Pharmacology, Sriram Chandra Bhanja Medical College and Hospital, Cuttack, IND

**Keywords:** scaly papule, compromised immunity, dermatology life quality index, psoriasis area severity index, plaque psoriasis

## Abstract

Background and objectives: In the Indian subcontinent, psoriasis cases have skyrocketed in the last decade. Dry and hot weather aggrandizes the annual incidences. Nowadays, dermatologists harness methotrexate and apremilast to manage chronic plaque psoriasis. There needs to be more comparative studies on these drugs. The primary objective was change in Psoriasis Area and Severity Index (PASI) at six months from the baseline. Change in Dermatology Life Quality Index (DLQI) at six months from the baseline and incidences of adverse events served as the secondary objectives.

Methods: This randomized, open-label, 24-week study was executed in Srirama Chandra Bhanja (SCB) Medical College, Cuttack, India, from June 2021 to October 2022. The participants were randomized in a 1:1 ratio to receive tablets of either methotrexate 10-15mg weekly once or apremilast 10-30mg twice daily. Efficacy and safety analyses were performed at baseline, eight, 16, and 24 weeks. We used R software (version 4.1.1; R Foundation for Statistical Computing, Vienna, Austria) for data analysis.

Results: Seventy (82.3%) of 85 enrolled participants completed the study. The mean age of the study population was 41.08±5.17 years. Twenty-two (31.4%) of them were females. The median change in PASI from baseline was -37.25 (-39.00 to -34.25) for apremilast and -34.75 (-37.75 to -31.75) for methotrexate (p=0.006). The median change in DLQI from baseline was -19.50 (-22.00 to -17.00) for apremilast and -21.00 (-25.50 to -17.50) for methotrexate (p=0.079). No serious adverse events were noticed.

Conclusion: Apremilast was more effective than methotrexate in psoriasis treatment. The statistically significant difference was found only in PASI scores.

## Introduction

Dermatological disorders have mushroomed since the age of industrial development [1]. Globally, chronic plaque psoriasis cases have burgeoned in the last decade [2]. In the Indian subcontinent, the dry, hot weather and prolonged sun exposure pave the way for rising incidences of plaque psoriasis [3]. In India, the summer season behaves like a double-edged sword for patients with psoriasis. While sunlight and humidity help in mild psoriasis symptoms, air conditioning and chlorine can dry out the skin and trigger flare-ups [4]. The annual incidence of psoriasis in India, 0.44% to 2.2%, is higher than the global incidence of 0.44% to 1.02% [5]. This disease has bimodal distribution and slightly higher male preponderance (1.3-1.7:1) [2,6-7].

Methotrexate is prescribed ubiquitously by skin specialists to treat mild to severe plaque psoriasis, pustular psoriasis, and psoriatic arthritis, regardless of age and gender [8]. It impedes the dihydrofolate reductase (DHFR) activity and meddles with folic acid activation. By arresting DNA synthesis, it limits epithelial hyperplasia, buttresses the apoptosis of activated T cells, and inhibits neutrophil chemotaxis. Furthermore, it dampens the production of pro-inflammatory cytokines such as tumor necrosis factor α (TNF-α) and interleukin-1 (IL-1) [9,10]. Apremilast was approved for the treatment of plaque psoriasis by the United States Food and Drug Administration (US-FDA) in 2014 and the Drug Controller General of India (DCGI) in 2017 [11,12]. Two landmark studies, ESTEEM [13] and PALACE [14], evaluated the efficacy of these drugs in psoriasis treatment and provided inconsonant results. Lately, a meta-analysis advocated higher efficacy of apremilast than placebo in the long-term management of psoriasis [15].

Since none of the previous studies have provided an irrefutable verdict regarding these two drugs, we outlined this study to determine the efficacy and safety of apremilast versus methotrexate monotherapy for 24 weeks in patients with chronic plaque psoriasis.

## Materials and methods

This prospective, interventional, two-arm, parallel-group, active-controlled, randomized, open-label, 24-week study was perpetrated from 6th June 2021 to 20th October 2022, involving patients with chronic plaque psoriasis in the departments of Pharmacology and Dermatology, Srirama Chandra Bhanja (SCB) Medical College, Cuttack, India. We obtained approval (IEC application no: 782 dated 04.06.2021) from the Institutional Ethics Committee of SCB Medical College, Cuttack, before enrolling the first participant. The patient information sheet, narrating the purpose of the study over and above the risk-benefit details for the participants, was provided in the local vernacular language, i.e., Odia, to each patient screened for eligibility. Written informed consent was obtained from all the study participants prior to their enrolment. This study was conducted per the Declaration of Helsinki and the principles of Good Clinical Practice.

Adult patients diagnosed with chronic plaque psoriasis with Psoriasis Area and Severity Index (PASI) [16] score ≥ 12, affected body surface area ≥ 10%, static Physician Global Assessment (PGA) score ≥ 2, and willing to provide written informed consent for participation were included in this study. The exclusion criteria were: persons previously diagnosed with pre-existing blood disorders like bone marrow hypoplasia, leukopenia, thrombocytopenia, anemia; those with a history of coagulopathies, thromboembolic disorders, recent stroke, myocardial infarction, deep vein thrombosis in last six months; those who were on any antiplatelet drugs, anticoagulants, or any iron supplementations; those with serum creatinine ≥ 2.5 mg/dl; persons with elevated liver enzymes or serum bilirubin levels; those who were on radiotherapy for psoriasis; those who were on any biologics for psoriasis in last six months; those who were on any topical agents for psoriasis in last two weeks; those who had prolonged exposure to sunlight; pregnant or lactating women.

We enrolled the patients who met the study criteria and randomly assigned them via block randomization in a 1:1 ratio to receive either apremilast or methotrexate for 24 weeks. We stratified the randomization based on gender (male or female) and age (≤ 40 or > 40 years) of the participant. The study was kept open-label. One group received tablet apremilast 10mg twice daily orally. Its dose was gradually increased to 30mg twice daily, which was then continued till the end of the study. The other group received tablet methotrexate 10mg orally once a week. Its dose was gradually increased to 15mg orally once a week, which was then continued till the end of the study. No cross-over of the study drugs was allowed. Participants in both groups were treated with paraffin cream as an add-on therapy.

At the baseline visit, we recorded sociodemographic parameters in a pre-prepared case record form. All participants underwent general physical and systemic examinations. Blood samples were collected to check hematological parameters and liver and kidney function tests. A thorough skin examination of the entire body was done to diagnose chronic plaque psoriasis and know the area of involvement. The privacy of all participants was maintained, and precautions regarding the confidentiality of their clinical details were taken care of. The PASI score and the Dermatology Life Quality Index (DLQI) score [17] for each participant were also noted at the baseline visit. After these proceedings, they received the drugs assigned to their groups free of cost. We asked for follow-up visits at eight, 16, and 24 weeks. The PASI and DLQI scores were noted at each follow-up visit. Those scores were clinically correlated for each participant. The incidence, severity, and seriousness of the adverse events of participants of the two study groups during the study duration were also noted. Both efficacy and safety assessments were performed as per the per-protocol (PP) analysis.

Based on the previous studies, the mean difference of PASI was considered for the calculation of the sample size of this study. Considering a mean difference of 15.0 in PASI from baseline, the standard deviation of 3.0, 62 patients (31 in each group) were required to detect a change in PASI with a power of 80% at a two-sided significance level of 0.05. Considering 10% drop-out or loss to follow-up, a sample size of 70 patients (35 in each group) was finalized for this study.

The normality of the data distribution was assessed prior to any analysis. The categorical data were presented as numbers (percentages). The continuous data were expressed as either mean ± standard deviation or median (inter-quartile range) depending on the normality of the data distribution. The between-group tests were done using the Mann-Whitney U test, and within-group tests were done using Friedman's test. A p-value < 0.05 was considered statistically significant. We used R software (version 4.1.1; R Foundation for Statistical Computing, Vienna, Austria) [18] for the data analysis and generation of the plots.

## Results

**Figure 1:**
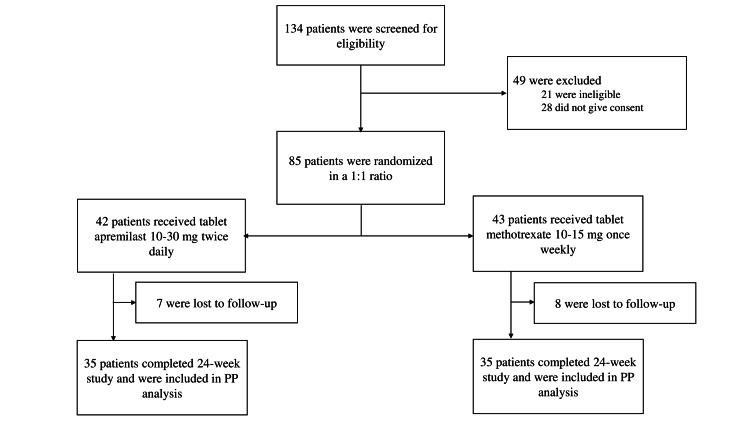
CONSORT diagram CONSORT: Consolidated Standard of Reporting Trials, PP analysis: Per-protocol analysis

One hundred thirty-four patients visiting Dermatology OPD, SCB Medical College, Cuttack, diagnosed with chronic plaque psoriasis, were screened for eligibility. Twenty-one were found ineligible, and 28 withdrew consent before enrolling in the study. A total of 85 patients meeting the study criteria were enrolled. They were randomly assigned in a 1:1 ratio to receive either of the study drugs. Of them, 42 received apremilast, and 43 received methotrexate for 24 weeks. A total of 15 participants were lost to follow-up (seven from the apremilast and eight from the methotrexate group, respectively). Finally, 35 participants in each group completed the study and were included in the efficacy and safety assessments per the PP analysis (Figure 1).

The baseline demographic and clinical parameters of the study population are shown in Table 1. The mean ages of the study participants of the apremilast and methotrexate group at the baseline visit were 40.86 ± 5.63 and 41.34 ± 5.31 years, respectively (p = 0.219). The median PASI scores of the study participants of the apremilast and methotrexate group at the baseline visit were 40.00 (37.75-42.25) and 40.00 (38.25-42.00), respectively (p = 0.734). The median DLQI scores of the study participants of the apremilast and methotrexate group at the baseline visit were 22.00 (17.25-25.50) and 23.00 (21.25-30.00), respectively (p = 0.372).

**Table 1:** Baseline sociodemographic and clinical parameters of the study population The categorical data were expressed as n (%). The continuous data were expressed as mean ± SD or median (interquartile range). PASI: Psoriasis Area and Severity Index, DLQI: Dermatology Life Quality Index.

	Apremilast (n =35)	Methotrexate (n =35)	p-value
Age (years)			
Mean ± SD	40.86 ± 5.63	41.34 ± 5.31	0.219
> 40 years	21 (60.00%)	16 (45.71%)	0.112
≤ 40 years	14 (40.00%)	19 (54.29%)	
Gender			
Male	23 (65.71%)	25 (71.42%)	0.273
Female	12 (34.29%)	10 (28.58%)	
Disease duration			
> 5 years	9 (25.71%)	10 (28.58%)	0.594
≤ 5 years	26 (74.29%)	25 (71.42%)	
Socioeconomic status			
Low	21 (60.00%)	20 (57.14%)	0.631
Middle	14 (40.00%)	15 (42.86%)	
Haemoglobin (g/dl)	12.37 ± 1.03	12.14 ± 0.98	0.568
Serum creatinine (mg/dl)	0.73 ± 0.17	0.86 ± 0.23	0.103
PASI	40.00 (37.75-42.25)	40.00 (38.25-42.00)	0.734
DLQI	22.00 (17.25-25.50)	23.00 (21.25-30.00)	0.372

**Figure 2:**
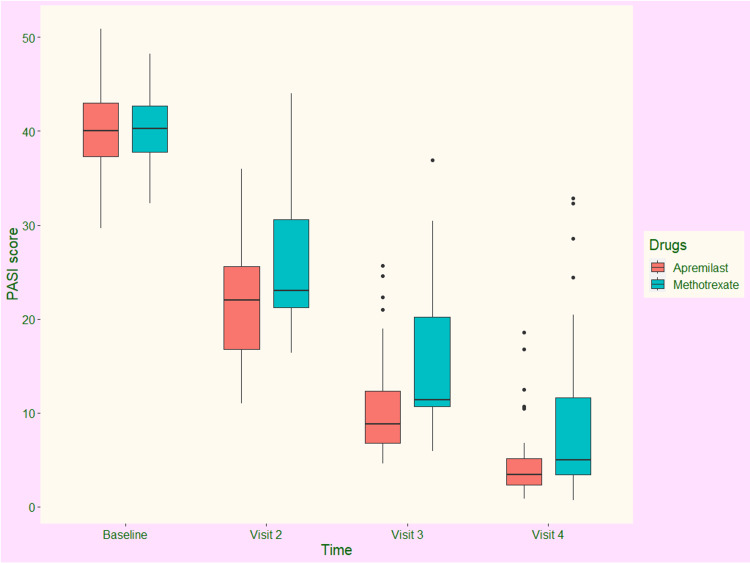
The PASI scores of the study population at various time points of assessment The box and whisker plots represent the PASI scores at baseline and the follow-up visits at eight, 16, and 24 weeks. PASI: Psoriasis Area Severity Index

The PASI scores of the study population at each time point of assessment are illustrated in Figure 2. At the baseline visit, the median PASI scores of participants receiving apremilast and methotrexate were 40.00 (37.75-42.25) and 40.00 (38.25-42.00), respectively (p = 0.734). During the second visit at eight weeks, the scores were reduced to 22.00 (17.00-25.50) and 23.00 (21.00-29.50), respectively (p = 0.151). After 16 weeks, the scores were further decreased to 9.00 (7.00-12.00) and 11.00 (10.50-19.50), respectively (p = 0.033). After 24 weeks of intervention, median PASI scores of apremilast and methotrexate groups were reduced to 3.50 (2.50-5.00) and 5.00 (3.50-11.00), respectively (p = 0.002). The median changes from the baseline of the two groups were -37.25 (-39.00 to -34.25) and -34.75 (-37.75 to -31.75), respectively (p = 0.006). The inter-group difference regarding the change in PASI score from baseline was clinically and statistically significant. It indicates higher efficacy of apremilast than methotrexate in treating chronic plaque psoriasis.

**Figure 3:**
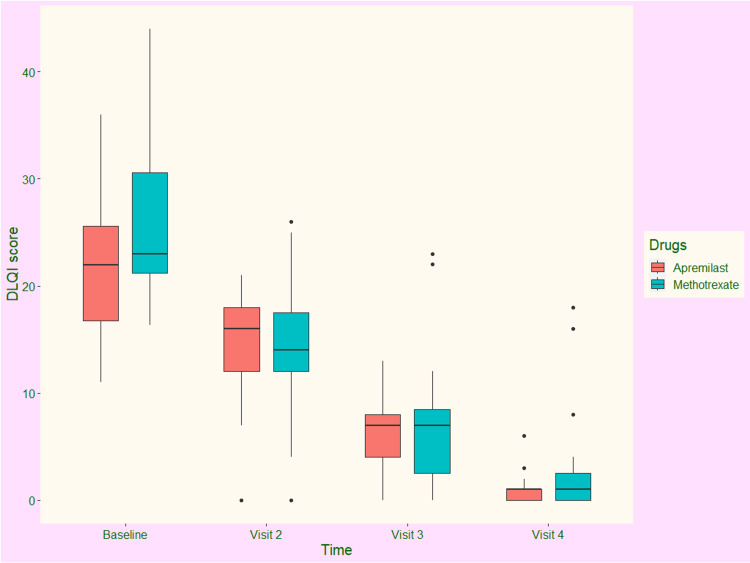
The DLQI scores of the study population at various time points of assessment The box and whisker plots represent the DLQI scores at baseline and the follow-up visits at eight, 16, and 24 weeks. DLQI: Dermatology Life Quality Index

The DLQI scores of the study population at each time of assessment are illustrated in Figure 3. At the baseline visit, the median DLQI scores of participants receiving apremilast and methotrexate were 22.00 (17.25-25.50) and 23.00 (21.25-30.00), respectively (p = 0.372). During the second visit at eight weeks, the scores were reduced to 16.25 (12.00-18.50) and 13.50 (12.00-18.00), respectively (p = 0.041). After 16 weeks, the scores were further decreased to 7.50 (5.00-8.50) and 7.50 (2.50-9.00), respectively (p = 0.117). After 24 weeks of intervention, median DLQI scores of apremilast and methotrexate groups were reduced to 1.50 (0.50-1.50) and 1.50 (0.50-3.00), respectively (p = 0.743). The median changes from the baseline of the two groups were -19.50 (-22.00 to -17.00) and -21.00 (-25.50 to -17.50), respectively (p = 0.079). Both drugs reduced the DLQI scores. However, the inter-group difference regarding the change in DLQI score from baseline was not statistically significant. It implies that the effects of the study drugs on the quality of life of participants with chronic plaque psoriasis were similar.

The incidences of adverse events in the study population are shown in Table 2. A total of 122 adverse events were noted in the participants receiving apremilast and methotrexate. The severity of each event was evaluated with a modified Hartwig scale. The majority of the events (87) were mild. Thirty-one events were moderate, and only four events were severe. Those severe events were diarrhea (one in the apremilast group), anemia (two in the methotrexate group), and nausea-vomiting (one in the methotrexate group). The seriousness of each adverse event was evaluated with Common Terminology Criteria for Adverse Events (CTCAE) version 5.0. All the events (122) were non-serious.

**Table 2:** Adverse events in the study population The severity of the adverse events was evaluated with Hartwig’s severity scale. The p-values were calculated using chi-square (χ2) or Fisher’s exact test. URTI: upper respiratory tract infections

	Apremilast (n =35)	Methotrexate (n =35)	p-value
Total	54	68	0.017
Severity of events			
Mild	41	46	0.038
Moderate	12	19	
Severe	1	3	
Individual events			
Diarrhea	12	6	0.021
Nausea-vomiting	9	11	0.053
URTI	10	4	0.009
Tension headache	7	1	0.013
Anaemia	2	18	< 0.001
Deranged liver enzymes	6	15	0.004
Rashes	8	13	0.019

## Discussion

This prospective, randomized, open-label study focused on the efficacy and safety of apremilast versus methotrexate after 24 weeks of intervention in patients with chronic plaque psoriasis. One group received a tablet of apremilast 10mg twice daily orally. Wherever required, the dermatologist gradually increased its dose to 30mg twice daily, which continued until the end of the study. The other group got a tablet of methotrexate 10mg orally once a week. If needed, the physician slowly increased its dose to 15mg orally once weekly. This study ascertained that apremilast was more efficacious than methotrexate in treating chronic plaque psoriasis. The effect on the quality of life of the affected individuals was similar to both drugs. Most of the adverse events were mild. Neither any serious events nor any mortality was reported.

After 24 weeks of intervention, median PASI scores of apremilast and methotrexate groups were reduced to 3.50 (2.50-5.00) and 5.00 (3.50-11.00), respectively (p = 0.002). The median changes from the baseline of the two groups were -37.25 (-39.00 to -34.25) and -34.75 (-37.75 to -31.75), respectively (p = 0.006). These findings were consistent with a meta-analysis by Puig et al. [15]. Regarding DLQI scores, the median changes from the baseline of the two groups were -19.50 (-22.00 to -17.00) and -21.00 (-25.50 to -17.50), respectively (p = 0.079). Both drugs reduced the DLQI scores. However, the inter-group difference regarding the change in DLQI score from baseline was not statistically significant [13,14].

Our study was strengthened by block randomization with stratification, frequent follow-up visits, and using an active drug as a comparator. However, there were a few limitations to our study as well. Firstly, a smaller sample size, probably because of the multitudinous eligibility criteria of the study and the global pandemic. Secondly, an open-label study design could have created room for recall bias, mainly during the safety assessments. Thirdly we could not gather extensive data regarding co-morbidities and other concomitant medications. We did not assess the effects of those drugs.

## Conclusions

We conclude that apremilast was more efficacious and safer in treating chronic plaque psoriasis than methotrexate. The quality of life was handled similarly by both drugs. We suggest further studies with a more heterogeneous study population to have one's eye on the long-term effects of these drugs on the general population.
